# Immuno-hematological parameters among adult HIV patients before and after initiation of Dolutegravir based antiretroviral therapy, Addis Ababa, Ethiopia

**DOI:** 10.1371/journal.pone.0310239

**Published:** 2024-10-31

**Authors:** Ayantu Gudina, Moges Wordofa, Fekadu Urgessa

**Affiliations:** 1 Maychew Health Center, Gulale Sub City, Addis Ababa, Ethiopia; 2 Department of Medical Laboratory Sciences, College of Health Sciences, Addis Ababa University, Addis Ababa, Ethiopia; University of Health and Allied Sciences, GHANA

## Abstract

**Background:**

Immuno-hematological abnormalities are common among HIV infected individuals as well as patients with highly active antiretroviral therapy (HAART). However, the immuno-hematological outcome of Dolutegravir based antiretroviral therapy (ART) usage is not well investigated.

**Objectives:**

To assess hematological and immunological parameters among adult HIV patients before and after initiation of Dolutegravir based ART regimen at St. Peter Specialized Hospital, Addis Ababa, Ethiopia.

**Methods:**

A cross-sectional study was conducted from May to July 2021 at St. Peter Specialized Hospital among adult HIV patients. A total of 422 HIV patients on Dolutegravir based ART (combination of Dolutegravir/lamivudine/tenofovir disoproxil fumarate (DTG/3TC/TDF)) for a minimum of 3 months were selected using convenient sampling methods. Socio-demographic as well as clinical data of the participants was obtained using pre-tested structured questionnaires and a review of medical records. Hematological parameters such as CBC was obtained using Beckman coulter automated hematology analyzer and immunological parameters such as CD4 count were determined using BD FACS presto. Statistical analysis of the data was done using SPSS version 21. Paired t-test was used to compare dependent variables before and after initiation of the new HAART and binary logistic regression was used to determine predictors of immuno-hematological abnormalities. P-value < 0.05 was considered as statistically significant.

**Results:**

Of 422 adult HIV patients, about 273(64.7%) were females. The mean age of study participants was 42.2 years (±10.4SD). The mean white blood cell (WBC) count, red blood cell (RBC) count, hemoglobin (Hb), platelet distribution width (PDW), CD4 count, as well as lymphocyte percentage, neutrophil percentage, and platelet counts (PLT) were increased significantly(P<0.05) after 3 months of the Dolutegravir based therapy. While, red cell distribution width (RDW) and mean cell hemoglobin (MCH) were decreased (P<0.05) after the treatment. Other hematological parameters such as mean cell volume (MCV), hematocrit (HCT), mean cell hemoglobin concentration (MCHC), mean platelet volume (MPV) and platelet distribution width (PDW) showed no significant change. On the other hand, the most common hematological abnormalities identified after the new HAART were anemia (12.1%); followed by Leucopenia (11.3%), neutropenia (6%), and thrombocytopenia (4%). Anemia was associated with female sex (AOR = 7.8, 95% CI: 1.9–32.2, P<0.005) and WHO clinical stage III/IV (AOR = 16, 95% CI: 10.63–66.46, P<0.01).

**Conclusion:**

There was a significant change in certain immuno-hematological parameters such as WBC count, RBC count, PLT count, Hb, PDW, CD4 count, lymphocyte and neutrophil percentage after initiation of the Dolutegravir based therapy. Anemia was the most common hematological abnormality. Further studies are required to fully comprehend the outcome of the new treatment regimen on immuno-hematological parameters.

## Introduction

HIV continues to be one of the world’s most serious public health concerns. Since the beginning of the HIV epidemic, more than 79 million people infected with the virus, and over 36 million have died. Globally, 37.7 million people were living with HIV at the end of 2020. An estimated 0.8% of adults aged 15–49 years worldwide are living with HIV, of which nearly 1 in every 25 adults (3.6%) living with HIV accounts African region which remains most severely affected [1]. Of adults living with HIV, 74% of them were accessing antiretroviral therapy as of June 2021 [2]. In Ethiopia, 690,000 people were living with HIV and the percentage of people living with the virus among adults was 0.9% in 2020 [3].

HIV infection is a multisystem disease, with hematological abnormalities amongst the most common pathological manifestations of the infection. Anemia, leukopenia, and thrombocytopenia are the most common abnormalities reported among HIV patients [4]. These abnormalities have been documented to be the second most common cause of morbidity and mortality in HIV patients [5]. Recent studies indicated that the virus involves almost all of the blood cell lineages and 63% of HIV patients had one or more cytopenia with anemia as the most common hematologic abnormality reported among the patients. However, leukopenia, neutropenia, lymphopenia, and thrombocytopenia have also been indicated [6–8]. The reasons for these derangements remain complex and multifactorial. HIV infects multipotent hematopoietic progenitor cells and establishes latent cellular reservoirs, disturbs the bone marrow microenvironment, and also causes immune deregulation [9, 10].

Anemia is the earliest and most common hematologic abnormality; affecting 60–90% of patients in the late-stage disease with lower CD4 count [11, 12]. A recent report indicates thrombocytopenia is the second most frequent haematological complication in nearly 3 to 40% of HIV infections and could occur at any stage of the infection [13]. Leukopenia is also a common occurrence, especially in patients with advanced clinical stage of the disease; amongst, neutropenia is the most common finding in 10–30% of the patients. Lymphopenia primarily involves CD4 T-helper cells and is considered the classic hallmark of the disease as it worsens with the progression of the disease [14, 15].

On the other hand, the use of antiretroviral drugs could also contribute to persistent hematopoietic suppression and subsequent hematologic abnormalities [16]. Treatments with many HAART therapy is also associated with a number of serious hematologic abnormalities with adverse effects that may ultimately limit the benefits of these treatments [17].

In Ethiopia, the impact of different HAART regimen on hematological parameters among HIV patients is still poorly documented. Despite the presence of few reports on the hematological profiles among adult HIV patients on ART in Ethiopia [5, 12, 17], which were exclusively done on prior ART regimens, the impact of the recently used ART regimen (Dolutegravir based combination treatment) on immuno-hematological parameters is not well evaluated. The country has recently shifted to Dolutegravir (DTG) based ART for newly HIV infected individuals, so that the treatment outcome of this ART regimen should be known.

Dolutegravir, a novel and well-tolerated integrase strand transfer inhibitors (INSTIs). With a median duration to maximum concentration ranging from 0.5 to 2 hours, it has shown to absorb quickly. More than 99% of the dolutegravir blood plasma concentrations are bound to albumin and alpha 1-acid glycoprotein, demonstrating the substantial protein binding of this drug. Dolutegravir exposure is enhanced when given with food, especially food rich in fat. Both ARV-experienced and naive patients, including those with first-generation INSTI resistance mutations, can benefit from its powerful antiretroviral activity. Dolutegravir has emerged as one of the most important components in the treatment of HIV infection because of its powerful efficacy, safety, simplicity in administration, and low medication interaction profile [18, 19].

Reports on the effect of DTG based ART on hematological parameters were very limited. A case study on a 56 years old Japanese man diagnosed with HIV showed severe thrombocytopenia during DTG-based ART [20]. Additionally, a study was conducted to evaluate the effects of dolutegravir (2.5–20 g/mL) on a number of pro-inflammatory activities of neutrophils isolated from the blood of healthy, adult humans. The exposure to dolutegravir alone caused the abrupt, dose-related, and significant generation of reactive oxygen species, which was attenuated by the addition of the Ca2+-chelating agent [21].

This study aimed to assess the effect of the new combination HAART on immuno-hematological parameters among HIV patients. This will enable physicians to consider the pros and cons of the treatment as well as for a more reliable interpretation of hematology laboratory findings.

## Methodology

### Study design and setting

Institution based cross-sectional study was conducted from May 1 to July 30, 2021, at St. Peter Specialized Hospital; which is one of the federal referral hospitals in Addis Ababa, Ethiopia. The hospital was established in 1953 as a public hospital. It has more than 300 beds and gives different inpatient and outpatient services to populations in the surrounding area. The hospital began providing a package of comprehensive care and treatment for HIV/AIDS in 2006 using two medical doctors and 6 nurses in ART unit. At the time of the study period, the hospital provided ART service for about 2367 HIV patients with counseling, caring, nutritional care, and routine laboratory monitoring [22].

### Sampling technique and sample size determination

A convenient sampling technique was used to select 422 adult HIV patients and a single population proportion was used to determine the sample size(N). By taking 50% prevalence of anemia (since there were no previous studies done on hematological outcome of Dolutegravir-based ART), with a margin of error(d) = 0.05 and 95% CI, the calculated sample size was 384. Considering the possibility of 10% non-compliance, the minimum required sample size was 422. Patients’ medical records were thoroughly reviewed for their treatment conditions and drug adherence. Those patients who had poor adherence or treatment interruption were also excluded from the study. In addition, pregnant women and patients who have been previously diagnosed with chronic diseases such as kidney failure, heart and liver disease as well as hematological malignancies were excluded due to the fact that these conditions would have a significant impact on hematological parameters.

### Data collection methods and procedures

We recruited patients who were on HAART follow-up during a data collection period; which was between May 1 to July 30, 2021. All the relevant data was accessible to the data collectors before and after data collection. Socio-demographic data of participants were obtained using interviewer administered questionnaires as well as the baseline clinical data such as WHO clinical stage, opportunistic infections (OIS), other co-morbidity, and HAART duration was collected data within the stated data collection period from medical records after checking the accuracy and completeness the data.

About 5ml of venous blood was collected using K_2_EDTA vacutainer test tube from each patient by experienced laboratory professionals working in the hospital. Beckman Coulter DXH800 automated hematology analyzer is used to determine complete blood count (total WBC count, absolute and relative count of each WBC type, RBC, and platelet count). CD4 count was determined using BD FACS presto. A blood sample was collected as per established sample collection guidelines and maintained storage condition protocol of the hospital until processing. Quality control (low, normal, and high) assay for complete blood count (CBC), obtained from Coulter Hematology analyzer manufacturer along with patients’ samples was run. A standard operating procedure (SOP) for test parameters and testing machine was made from the manufacturer’s instructions of Beckman Coulter (DxH800).

### Operational definitions

**Dolutegravir (DTG) based ART** was a newly initiated 1J based HAART regimen, which is the combination of DTG/3TC/TDF.

**Immunosuppression** is defined based on the CD4 count <500 cells/μl as per the WHO guideline.

**Anemia, leucopenia, neutropenia, and thrombocytopenia** were defined based on the established St. Peter Specialized Hospital laboratory reference range and according to WHO hemoglobin cutoffs. Accordingly, for females, anemia was defined as Hb concentration < 12.0 g/dl (11.0–11.9 g/ dL = mild, 8.0–10.9 g/ dL = moderate, and < 8.0 g/dL = severe) while for males, anemia was defined as Hb concentration <13 g/dL (11.0–12.9 g/dL as mild; 8.0–10.9 g/ dL as moderate, and < 8.0 g/dL as severe) [23].

A platelet count of 150–450×10^3^/mm^3^ is considered normal and thrombocytopenia is defined as a total platelet count < 150 × 10^3^/mm^3^ and white blood cell count (WBC) of 4–11× 10^3^/mm^3^ is considered normal and leucopenia as defined as total WBC count < 4 × 10^3^/mm^3^.

### Statistical analysis

After the collected data was cleaned and verified, it was analyzed using SPSS version 21. Appropriate statistical method like t-test was applied to compare differences in immuno-hematological outcomes before and after the new HAART initiation and logistic regression analysis was used to determine the associations of established risk factors for immunologic and hematologic abnormalities. Statistical significance was considered at P< 0.05 with 95% CI.

### Ethical clearance

This study was approved by the Research and Ethics Review Committee of the Department of Medical Laboratory Sciences, College of Health Sciences, Addis Ababa University; with a protocol number, DRERC/594/21/MLS. In addition, an official letter of permission was obtained from St. Peter Specialized Hospital. Written informed consent was obtained from all participants after they had been briefed and understood the objective of the study. Confidentiality of the data was also maintained throughout the study.

## Results

### Socio-demographic characteristics of study participants

In this study, a total of 422 adult HIV patients were included and the median (±SD) age of the participants was, 42.2(±10.4) years and most of them, 273 (64.7%) were females. The majority of participants, 255(60.4%) and 183(43.4%) had low-income status and married, respectively (Table 1).

**Table 1:** Socio-demographic characteristics of HIV patients with a Dolutegravir based ART at St. Peter Specialized Hospital, Addis Ababa, Ethiopia, 2021(N = 422).

Variables	Category	N (%)
Age (years)	18–34	81(19.2)
	35–45	199(47.2)
	>45	142(33.6)
	Mean±SD	42.2±10.4
Sex	Male	149(35.3)
	Female	273(64.7)
Marital status	Single	66(15.6)
	Married	183(43.4)
	Divorced	112(26.5)
	Widowed	61(14.5)
Income status *	Low(<500ETB)	255(60.4)
	Medium(3001-5000ETB)	127(30.1)
	High(>5000ETB)	40(9.5)

* Income status category was based on Ethiopia Demographic and Health Survey (EDHS), 2019

ETB: Ethiopian Birr (1ETB = 0.0229USD)

SD = standard deviation.

### Baseline clinical characteristics of study participants

Before shifting to the new HAART (Dolutegravir based ART), the majority (64.7%) of study participants were treated with TDF- based regimens and 269(63.7%) of patients were on WHO clinical stage I and II (Table 2).

**Table 2:** Baseline clinical characteristics of HIV patients on ARV before initiation of Dolutegravir based regimen at St. Peter Specialized Hospital, Addis Ababa, Ethiopia, 2021(N = 422).

Variables	Category	N (%)
WHO stages	I-II	269 (63.7)
	III-IV	153(36.3)
HAART type	AZT based	149(35.3)
	TDF based	273(64.7)
CD4 count (cells/μl) (mean±SD)	435.56±35.5	
Hb(g/dl) (mean±SD)	14.3±2.69	
HAART duration (months) (mean±SD)	12.07± 6.25	

### Immuno-hematological parameters outcome among study participants after Dolutegravir based ART

Paired t-test was applied to realize the mean variation of immuno-hematological outcome among study participants before and after the new HAART. After shifting to the new treatment for >3 months, the mean WBC count, RBC count, Hb, PDW, CD4 count, as well as LYM (%), NEU (%), and PLT count were increased significantly(P<0.05). However, RDW and MCH were decreased (P<0.05) (Table 3).

**Table 3:** Immuno-hematological parameters of adult HIV patients before and after Dolutegravir based ART at St. Peter Specialized Hospital, Ethiopia.

Parameters	(mean± SD) values		P-value
	Before the new HAART	3 months after The new HAART initiation	
CD4 count (cells/μl)	435.56±235.50	539.63±236.53	<0.001
WBC count (10 ^3^/mm^3^)	5.2 ±6.29	6.43±3.59	<0.001
RBC count (10 ^6^/mm^3^)	4.35±0.72	7.9±0.47	<0.05
Hb(g/dl)	14.43±2.69	15.1±2.68	<0.05
HCT (%)	42.8±2.09	45.1±6.97	0.15
PLT count (10 ^3^/mm^3^)	250.65±70.14	260.14± 89.93	<0.05
LYM (%)	33.13±12.58	35.82±11.42	<0.001
MON (%)	5.44 ± 3.32	7.98±3.2	<0.001
NEU (%)	50.64± 13.01	56.65±12.25	<0.001
MCV (fl)	96±13.02	94.28±9.68	0.38
MCH (pg)	35.66±5.4	31.65±2.77	<0.05
MCHC(g/dl)	34.42±2.08	36.45±2.6	0.11
RDW (%)	17.11±11.52	14.74 ±9.66	<0.05
MPV (fl)	9.90 ±1.62	9.78 ±6.79	0.901
PDW (%)	12.38±6.13	14.67±1.98	<0.05

As shown in Table 3 above, the mean WBC count after the new treatment initiation was significantly increased compared to the mean before the treatment. Thus, the change was evaluated by stratifying it into four HAART duration categories. Accordingly, as shown in Table 4, the mean WBC count was highest for the treatment duration of >18 months (7.1x10^3^/mm^3^) compared to the rests;12–18 months (6.4x10^3^/mm^3^),6–12 months (6.3x10^3^/mm^3^) and < 6 months (5.75x10^3^/mm^3^); indicating that, the mean WBC increases with increasing duration on HAART.

**Table 4:** Immuno-hematological parameters among adult HIV patients after Dolutegravir based ART, for the duration at St. Peter Specialized Hospital, Addis Ababa, Ethiopia, 2021.

	Before HAART		After a new HAART			F	P
Parameters			Duration of the treatment				
		<6 Months	6–12 Months	12-18Months	>18Months		
CD4(cells/μl)	435.56±35	534.3±243.3	555.13±360.9	581.10±156	511.82±63	0.43	.725
WBC (10^3^/mm^3^)	5.2 ±6.29	5.75±4.78	6.30±1.54	6.42±1.62	7.1±5.7	2.73	.**043**
RBC (10^6^/mm^3^)	4.35±0.72	8.50 ±41	12.59±59.9	5.08±2.7	4.63±.61	1.10	0.34
Hb (g/dl)	14.43±2.6	16.25±11.03	14.29 ±1.61	15.02±2.26	14.62± 2.1	2.21	.086
HCT (%)	42.8±2.1	44.38 ±4.48	42.53 ±4.6	44.17±5.41	45.94± 5.3	1.52	.207
PLT (10^3^/mm^3^)	260.1±89.9	238.74±65.1	266.82±67.53	250.66±73.1	247.79±73	3.11	.**026**
LYM (%)	33.1+12.58	28.92±10.37	30.34±13.20	30.03+9.97	35.1±11.5	2.52	.057
MON (%)	5.44 ± 3.32	8.274±4.39	8.274±2.16	8.274 ±3.23	8.274±2.64	1.67	.171
NEU (%)	50.64±13.0	53.13±14.20	55.81±12.27	57.57±14.06	58.4±12.5	3.31	.**020**

In the current study, the mean LYM (%) of the study participants significantly increased after a minimum of 3 months of the new HAART initiation (P = <0.001) (Table 3). However, when the mean LYM (%) was further compared after stratifying the new treatment duration into four categories, it was found to be statistically insignificant (P = 0.057)(Table 4).

The mean±SD of a platelet count of the study participants was, 250.65±70.14×10^3^/μl before starting the new HAART and significantly increased to 260.14± 89.93 ×10^3^/μl after the treatment (P< .001) as shown in Table 3. In addition, as illustrated in Table 4, this mean increment in platelet count across the treatment duration category was significantly different between groups (p = 0.026).

Moreover, the average NEU (%) was also significantly increased after three months of the treatment as compared to the one before the treatment (P<0.001) (Table 3). Moreover, there was a significant change in the average neutrophil percentage as the treatment duration increased (P = .020); indicating the improvements of neutrophil count over time (Table 4).

### Magnitude of hematological abnormalities before and after Dolutegravir based ART treatment

In our study, the magnitude of anemia before the new treatment was 24%; while it reduced to 12.1% after at least three months of the Dolutegravir based ART. Similarly, the magnitude of leucopenia before the new treatment initiation was 30.5% and decreased to 11.3% after the treatment. The change in the level of immunosuppression was observed, decreasing from 64.5% to 50.9% before and after the new treatment, respectively (S1 Fig).

### Anemia and its predictors among HIV patients after Dolutegravir based ART

In this study, predictors for anemia among patients with Dolutergravir based treatment were sex, WHO clinical stage, and CD4 count. Patients with WHO clinical stages three and four had increased odds of having anemia than those with stages one and two (AOR = 16,95%CI:10.63–66.46, P = 0.01). Similarly, females had 7.8 times increased risk of developing anemia than male participants (AOR = 7,8,95%CI:1.9–32.2, P = 0.04) (Table 5).

**Table 5:** Predictors of anemia among study participants after Dolutegravir based ART at St. Peter Specialized Hospital, Addis Ababa, Ethiopia, 2021.

Variables	Anemia		AOR (95%CI)	P-value
	Yes, N (%)	No, N (%)		
Sex:				
Female	39(14.3)	234(85.7)	7.8(1.9–32.2)	.004
Male	12(8.1)	137(91.9)	1	
Age (years)				
18–34	9(11.1)	72(88.9)	1	0.37
35–45	29(14.6)	170(85.4)	1.1(.43–2.79)	.83
>45	13(9.2)	1129(90.8)	.61(.30–1.56)	.36
CD4count				
>200	5(1.4)	3354(98.6)	1	
<200	46(73)	17(27)	32 (46.3–98.5)	.0001
WHO Stage				
I-II	8(3)	261(97)	1	
III-IV	43(28)	110(72)	16(10.63–66.46)	.001

### CD4 outcome A

#### CD4 outcome after a Dolutegravir based ART and its associated factors

This study also indicated that, factors associated with immune suppression after at least three months of new HAART usage were: sex, baseline CD4 count, and income status of the participants. Female participants had 2.65 times increased risk of immune-suppression than males after at least 3 months of new HAART initiation (AOR = 2.65,95%CI:1.47–4.79, P = 0.01). The study also revealed that participants with <200 baseline CD4 count had 10.8 times increased risk of immune suppression after the treatment (AOR = 10.8,95%CI: 4.67–24.67, P = 0.001) as compared to baseline CD4 count ≥500. Similarly, participants with a baseline CD4 count between 200 to 349 had 3.27 increased odds of immune-suppression after at least 3 months of the treatment (AOR = 3.27,95%CI: 1.43–7.47, P = 0.05) than those with a baseline CD4 count of ≥500.

Finally, participants with low (AOR = 6.2, 95%CI:1.96–19.95, P = 0.02) and medium (AOR = 2.5, 95%CI:1.31–4.77) income had 6.2 and 2.5 times, respectively increased odds of immune-suppression after 3 months of treatment than those with high income (Table 6).

**Table 6:** Predictors of immune suppression after Dolutegravir based ART at St. Peter Specialized Hospital, Addis Ababa, Ethiopia, 2021.

	Immune suppression		AOR (95%CI)	P-Value
	Yes, N (%)	No, N (%)		
Sex:				
Male	93(62.4)	56(37.6)	1	
Female	122(44.7)	151(55.3)	2.65(1.47–4.7 9)	.0
Age (Years)				
18–34	41(50.6)	40(49.4)	1	.31
35–45	101(50.8)	98(49.2)	1.6(.810–3.313	
>45	73(51.4)	69(48.6)	1.75(.809–3.78)	
Base line CD4 category				
≥500	29(19.2)	122(80.8)	1	
350–499	46(47.9)	50(52.1)	-	
200–349	90(81.1)	21(18.9)	3.27(1.43–7.47)	.005
<200	50(78.1)	14(21.9)	10.8(4.67–24.67)	.001
Income Status				
High	7(25.9)	20(74.1)	1	
Medium	43(41.3)	61(58.7)	2.5(1.31–4.77)	.005
Low	165(56.7)	126(43.3)	6.2(1.96–19.95)	.002
Opportunistic infections				
No	170(51.8)	158(54.3)	1	
Yes	45(47.9)	49(52.1)	0.85(.539–1.351)	.499

## Discussion

This study investigated the immuno-hematological outcomes of adult HIV patients on newly initiated Dolutegravir based ARV treatment at St. Peter Specialized Hospital. After at least 3 months of the new treatment, many hematological parameters such as, WBC, RBC, Hb, PLT, %NUE, %MON, %LYM, and CD4 count have shown improvements. On the other hand, RDW and MCH significantly decreased.

This study showed that the white cell counts significantly improved after a change in the treatment regimen and this is in line with findings of Talargia F *et al*., 2021 [24] study done in North-East Ethiopia as well as a study by Princy JJ et al.,2021 [25] in India; which reported a significant increment in WBC after initiation of the combination therapy. However, contrary to this finding, a study done at Debre Tabor Comprehensive Specialized Hospital, Ethiopia, showed a decrease in the mean WBC (6.5±2.4 10^9^/L vs5.1±1.210^9^/L) compared to the one before HAART initiation [26]. The discrepant finding could be due to, the difference in HAART regimen and study population. In addition, patients may have infections that lead to increase in WBC before ARV therapy, and the treatment may also improve immunity; minimizing the possibility of individuals who initiated HAART being infected by OIS [27].

The current study indicated that, the percentage of neutrophils and monocyte, had significantly increased after the initiation of the new treatment, and a study in Rome, Italy also showed there was an improvement in the neutrophils count after receiving ART. This may be due to the fact that, after new ARV treatment, there is improvement in immunity against OIS that suppresses the bone marrow neutrophil and monocyte function and expression [28].

In our study, the mean lymphocyte also showed a significant increment after 3 months of the new ART initiation. This is in line with the study conducted in India [4] and in Rome [28]. However, it is contraindicated with other reports [24, 29]. The current study showed improvement in mean RBC, HCT, MCHC, and decrement in RDW after ART initiation. A similar finding is reported in other studies [30–32]. This could be due to the positive feedback of the new HAART that helps in the decline of viral load, decreased release of immature red blood cells, decreased destruction of red blood cells, and improved erythropotein(EPO) response [33]. However, this finding is contradicted with the finding of Damtie S *et al*., 2021, Ethiopia [26] and Iran [31].

In this study, the mean platelet count was 250.65x10^3^/μl, and 260.14x10^3^ /μl before and after the new HAAR, respectively. In line with this, other studies also reported incremental in mean platelet count after HAART initiation [31, 34–37]. This might be due to the beneficial effect of active ART in reducing the frequencies of thrombocytopenia [36, 38].

From this finding, the magnitude of anemia was found to be 24% and 12.1% before and after the new HAART initiation, respectively. Moreover, the predictors of anemia were sex (female) and Low CD4 (<200count). This is concordant with the findings of Harris *et al*., in 2008, which revealed the decreased magnitude of anemia before ART, 35% to 26% after the initiation with 21.4%, 3.7%, 0.4% of mild, moderate, and severe anemia respectively [39]. Other studies also indicated anemia is associated with, low CD4 count(<200) and advanced clinical stage (III/IV) of HIV [12, 17, 25, 30, 40]. The decreased anemia after ARV treatment is due to the positive effect of active ART in reducing the frequencies of anemia, and also the positive feedback of the new ARV treatment that helps in the decline of viral load, decreased destruction of red blood cells either by a virus infection,suppression of OIS that lead to anemia [27].

In the current study, thrombocytopenia was noticed in about 6% of patients before the new HAART treatment and 4% after the treatment. This is in line with a study conducted in Beijing Ditan Hospital, China in 2021 on thrombocyte abnormalities among HIV positive patients before and after the initiation of ART and showed the prevalence was 2.65% among ART-naive patients [38] and in Addis Ababa, Ethiopia, the prevalence of thrombocytopenia after HAART was 4.1% [41]. The reduction of thrombocytopenia could be due to decreased infection of megakaryocytes by the HIV virus after HAART, reduction of immune-mediated destruction of platelets by antibodies as well as cross-reacting antibodies that are targeted against HIV proteins, elimination of OIS that cause marrow suppression which led to thrombocytopenia after effective ARV therapy [42]. Contrary to the present study, a study conducted in Japan reported severe thrombocytopenia during dolutegravir (DTG) containing antiretroviral therapy. After initiation of DTG, the platelet count was decreased to ≤50, 000/μl. This could be due to the direct infection of megakaryocytes by HIV virus or co-infection with another virus, opportunistic infections, and myelosuppression effect of medication [21].

Our study also showed that, the prevalence of leukopenia was 30.5% and 14% before and after new HAART, respectively. This finding is concordant with a study conducted by Thulasi *et al*., 2016 showed the magnitude and severity of leucopenia was found to be low in patients after new ARV treatment [43]. However, Contrary to current findings, a study conducted in Ghana reported an increased prevalence of leukopenia after initiation of HAART [44]. This abnormality could have resulted from the use of a HAART regimen, which can cause a suppression of bone marrow production and cytotoxicity of T-cells, eventually decreasing the survival of T-cells. But, in this study, leukopenia decreased by more than 50% after new ART initiation. This could be due to differences in HAART types; other previous studies used the first line of ART includes a combination of non-nucleoside reverse transcriptase inhibitors (NNRTI’s) and nucleoside reverse transcriptase inhibitors (NRTI’s) based but the current study was based on integrase strand transfer inhibitors (INSTIs) or Dolutegravir (DTG).

In our study, neutropenia was 11.3%, before new HAART and 6% after the treatment. Similarly, a study done in North-East Ethiopia, in 2020, showed a decrement in the magnitude of neutropenia; which was 7.0%, before the initiation of ART and 1.1% after the treatment [24]. Other findings have also reported a decrease in the prevalence of neutropenia after ARV therapy [4, 45, 46]. The higher magnitude of neutropenia before HAART initiation was due to increased marrow suppression by OIS which in turn altered neutrophil production, decreased amounts of granulocyte colony-stimulating factor, and accelerated apoptosis [14]. However, contrary to the present finding, a study in Gondar, Ethiopia reported 14.5% of neutropenia before HAART and 28.3% after initiation of the treatment [30].

In this study, the mean CD4+ T cell count was significantly increased from 435.56 cells/mm^3^ before new HAART initiation to 539.63 cells/mm^3^ after the treatment. This is in line with the cross-sectional study done in North-East Ethiopia, in 2021, which showed CD4+ T cell counts of 264.75 cells/mm^3^ and 544 cells/mm^3^, before and after HAART initiation, respectively [24]. Other studies have also reported the improvement in mean CD4 count after HAART [26, 47–50]. The increment in mean CD4 count is due to the fact that, the HAART plays a vital role in the depletion of the viral load which in turn contributes to the immune recovery and efficacy of new ART treatment in improving the CD4 count even within three months of beginning treatment.

The predictors of detected immune suppression after at least three months of new HAART usage were: sex, baseline CD4 count<200 cells/mm^3^and income status of the participants. Female participants had 2.65 times increased risk of immune-suppression than males after at least 3 months of new HAART initiation (AOR = 2.65, 95%CI: 1.47–4.79, P = 0.01). Participants with <200 baseline CD4 count had 10.8 times increased risk of immune-suppression after at least 3 months of new ARV treatment (AOR = 10.8,95%CI: 4.67–24.67, P = 0.001) as compared to baseline CD4 count ≥500. Participants with low (AOR = 6.2,95%CI:1.96–19.95, P = 0.02) and medium (AOR = 2.5,95%CI:1.31–4.77) income had 6.2 and 2.5 times, respectively increased odds of immune-suppression after 3 months of treatment than those with high income. This is consistent with the study done by Vemula et al., 2016, India, who reported that, for those who were initiated with ART of CD4 count < 350 cells/mm^3^, the count was increased by a mean of 180.28 cells/mm^3^ after 6 months of HAART use. In this study, female participants were also shown to have more improvement in CD4 count than males [47] and a study by Rajasuriar R *et al*., in Australia reported a lesser but significant number of patients did not achieve CD4 T-cell counts >500cells/ml despite years of suppressive combination antiretroviral therapy (cART). Their finding also showed that clinical factors associated with CD4 T-cell recovery following long-term cART were, higher baseline CD4 T-cell counts (P = 0.001), younger age (P = 0.019), and treatment initiation with a protease inhibitor (PI)-based regimen vs. non-nucleoside reverse transcriptase inhibitor, NNRTI; (P = 0.043). The time taken to achieve a CD4 T-cell count >500 cells/ml despite long-term cART is prolonged in a subset of patients. Starting cART early with a PI-based regimen vs. NNRTI-based regimen is concomitant with more rapid recovery of a CD4 T-cell count >500 cells/ml [51]. In other studies, similar results were obtained, where lower CD4 outcome is related to lower baseline CD4 cell counts, longer HAART duration, and older age [49, 52–56]. However, our finding contradicts with study conducted in Peru to assess Predictors of CD4+ cell count response and of adverse outcomes among HIV-infected patients receiving HAART and the results showed that, patients with a lower CD4+ cell count at baseline and those starting HAART with a didanosine-based regimen had a higher increase in CD4+ cell count at six months [57]. The difference could be the difference in HAART type or regimen,duration, and population characteristics.

As a limitation, the viral load status of the patients was not considered to evaluate the effectiveness of Dolutegravir based ART. In addition, hematological and CD4 outcomes for each type of ARV treatment were not assessed.

## Conclusion

The mean CD4 count, RBC count, WBC count, neutrophil percentage, Platelets count, Hb, LYM (%), and HCT (%) significantly increased among study participants; Whereas, RDW and MCH significantly decreased after at least 3 months of Dolutegravir based ART. Moreover, the current finding indicated the mean WBC, LYM%, and PLT counts after the new HAART increased with increasing duration of the treatment.

The predictors of immune suppression of CD4 count <500 cells/μl after at least three months of the new HAART were; sex, baseline CD4 count, and income status of the patients.

## Supporting information

S1 Checklist STROBE statement—checklist of items that should be included in reports of observational studies. (DOCX)

S1 Fig Common immuno-hematological abnormalities among HIV patient. (TIF)
