# Supplementary material for: Immuno-hematological parameters among adult HIV patients before and after initiation of Dolutegravir based antiretroviral therapy, Addis Ababa, Ethiopia
Source: PLoS One. 2024 Oct 31;19(10):e0310239. doi: 10.1371/journal.pone.0310239 (PMC11527299; doi:10.1371/journal.pone.0310239)
Supplement: S1 Checklist — (DOCX) [file pone.0310239.s001.docx]

STROBE Statement—checklist of items that should be included in reports of observational studies

|  | Item No. | Recommendation | Page  No. | Relevant text from manuscript |
| --- | --- | --- | --- | --- |
| **Title and abstract** | 1 | (*a*) Indicate the study’s design with a commonly used term in the title or the abstract | 1-2 | Cross-sectional study was conducted from May to July, 2021 |
|  |  | (*b*) Provide in the abstract an informative and balanced summary of what was done and what was found | 1-2 | A cross-sectional study was conduct among a total of 422 HIV patients on Dolutegravir based ART. There was a significant change in certain immuno-hematological parameters after initiation of the Dolutegravir based therapy |
| Introduction | | | |  |
| Background/rationale | 2 | Explain the scientific background and rationale for the investigation being reported | 3-4 | In Ethiopia, the impact of different HAART regimen on hematological parameters among HIV patients is still poorly documented |
| Objectives | 3 | State specific objectives, including any prespecified hypotheses 3-4 |  | This study was aimed to assess the effect of the new combination therapy on hematological and immunological parameters among HIV patients |
| Methods | | | |  |
| Study design | 4 | Present key elements of study design early in the paper 4 |  | Institution based cross-sectional study was conducted from May 1 to July 30, 2021 |
| Setting | 5 | Describe the setting, locations, and relevant dates, including periods of recruitment, exposure, follow-up, and data collection | 4-5 | Institution based cross-sectional study was conducted from May 1 to July 30, 2021 at St. Peter specialized Hospital; which is one of federal referral hospital in Addis Ababa, |
| Participants | 6 | (*a*) *Cohort study*—Give the eligibility criteria, and the sources and methods of selection of participants. Describe methods of follow-up  *Case-control study*—Give the eligibility criteria, and the sources and methods of case ascertainment and control selection. Give the rationale for the choice of cases and controls  *Cross-sectional study*—Give the eligibility criteria, and the sources and methods of selection of participants | 5-6 | A convenient sampling technique was used to select 422 adult HIV patient and single population proportion was used to determine the sample size(N). We recruited patients who were on ART follow up during a data collection period. |
|  |  | (*b*) *Cohort study*—For matched studies, give matching criteria and number of exposed and unexposed  *Case-control study*—For matched studies, give matching criteria and the number of controls per case |  |  |
| Variables | 7 | Clearly define all outcomes, exposures, predictors, potential confounders, and effect modifiers. Give diagnostic criteria, if applicable |  |  |
| Data sources/ measurement | 8* | For each variable of interest, give sources of data and details of methods of assessment (measurement). Describe comparability of assessment methods if there is more than one group |  |  |
| Bias | 9 | Describe any efforts to address potential sources of bias | 5-6 | Patients who have been previously diagnosed with chronic disease such as kidney failure, heart and liver disease as well as hematological malignancies were excluded due to the fact that these conditions would have a significant impact on hematological parameters. In addition, pregnant HIV women, patients who had poor adherence or treatment interruption were also excluded from the study |
| Study size | 10 | Explain how the study size was arrived at |  |  |

| Quantitative variables | 11 | Explain how quantitative variables were handled in the analyses. If applicable, describe which groupings were chosen and why |  | 6-7 After the collected data was cleaned and verified, it was analyzed using SPSS version 21 |
| --- | --- | --- | --- | --- |
| Statistical methods | 12 | (*a*) Describe all statistical methods, including those used to control for confounding |  | 6-7 |
|  |  | (*b*) Describe any methods used to examine subgroups and interactions |  |  |
|  |  | (*c*) Explain how missing data were addressed | 5-6 | data collected from medical records after checking the accuracy and completeness the data. |
|  |  | (*d*) *Cohort study*—If applicable, explain how loss to follow-up was addressed  *Case-control study*—If applicable, explain how matching of cases and controls was addressed  *Cross-sectional study*—If applicable, describe analytical methods taking account of sampling strategy | 5 | single population proportion was used to determine the sample size(N) |
|  |  | (*e*) Describe any sensitivity analyses |  |  |
| Results | | | | |
| Participants | 13* | (a) Report numbers of individuals at each stage of study—eg numbers potentially eligible, examined for eligibility, confirmed eligible, included in the study, completing follow-up, and analysed | 7 |  |
|  |  | (b) Give reasons for non-participation at each stage | 5 |  |
|  |  | (c) Consider use of a flow diagram |  |  |
| Descriptive data | 14* | (a) Give characteristics of study participants (eg demographic, clinical, social) and information on exposures and potential confounders | 7-8 | Socio-demographic characteristics of HIV patients with a Dolutegravir based ART |
|  |  | (b) Indicate number of participants with missing data for each variable of interest |  |  |
|  |  | (c) *Cohort study*—Summarise follow-up time (eg, average and total amount) |  |  |
| Outcome data | 15* | *Cohort study*—Report numbers of outcome events or summary measures over time |  |  |
|  |  | *Case-control study—*Report numbers in each exposure category, or summary measures of exposure |  |  |
|  |  | *Cross-sectional study—*Report numbers of outcome events or summary measures |  |  |
| Main results | 16 | (*a*) Give unadjusted estimates and, if applicable, confounder-adjusted estimates and their precision (eg, 95% confidence interval). Make clear which confounders were adjusted for and why they were included | 7-15 |  |
|  |  | (*b*) Report category boundaries when continuous variables were categorized |  |  |
|  |  | (*c*) If relevant, consider translating estimates of relative risk into absolute risk for a meaningful time period |  |  |

Continued on next page

Continued on next page

| Other analyses | 17 | Report other analyses done—eg analyses of subgroups and interactions, and sensitivity analyses |  |  |
| --- | --- | --- | --- | --- |
| Discussion | | | | |
| Key results | 18 | Summarise key results with reference to study objectives |  | 15-20 |
| Limitations | 19 | Discuss limitations of the study, taking into account sources of potential bias or imprecision. Discuss both direction and magnitude of any potential bias |  | 20 As a limitation, viral load status of the patients was not considered to evaluate the effectiveness of Dolutegravir based ART. In addition, hematological and CD4 outcome for each type of ARV treatment were not assessed |
| Interpretation | 20 | Give a cautious overall interpretation of results considering objectives, limitations, multiplicity of analyses, results from similar studies, and other relevant evidence |  | 20 |
| Generalisability | 21 | Discuss the generalisability (external validity) of the study results |  |  |
| Other information | |  | | |
| Funding | 22 | Give the source of funding and the role of the funders for the present study and, if applicable, for the original study on which the present article is based |  |  |

*Give information separately for cases and controls in case-control studies and, if applicable, for exposed and unexposed groups in cohort and cross-sectional studies.

**Note:** An Explanation and Elaboration article discusses each checklist item and gives methodological background and published examples of transparent reporting. The STROBE checklist is best used in conjunction with this article (freely available on the Web sites of PLoS Medicine at http://www.plosmedicine.org/, Annals of Internal Medicine at http://www.annals.org/, and Epidemiology at http://www.epidem.com/). Information on the STROBE Initiative is available at www.strobe-statement.org.
