# Supplementary figures and images for: Immuno-hematological parameters among adult HIV patients before and after initiation of Dolutegravir based antiretroviral therapy, Addis Ababa, Ethiopia
Source: PLoS One. 2024 Oct 31;19(10):e0310239. doi: 10.1371/journal.pone.0310239 (PMC11527299; doi:10.1371/journal.pone.0310239)

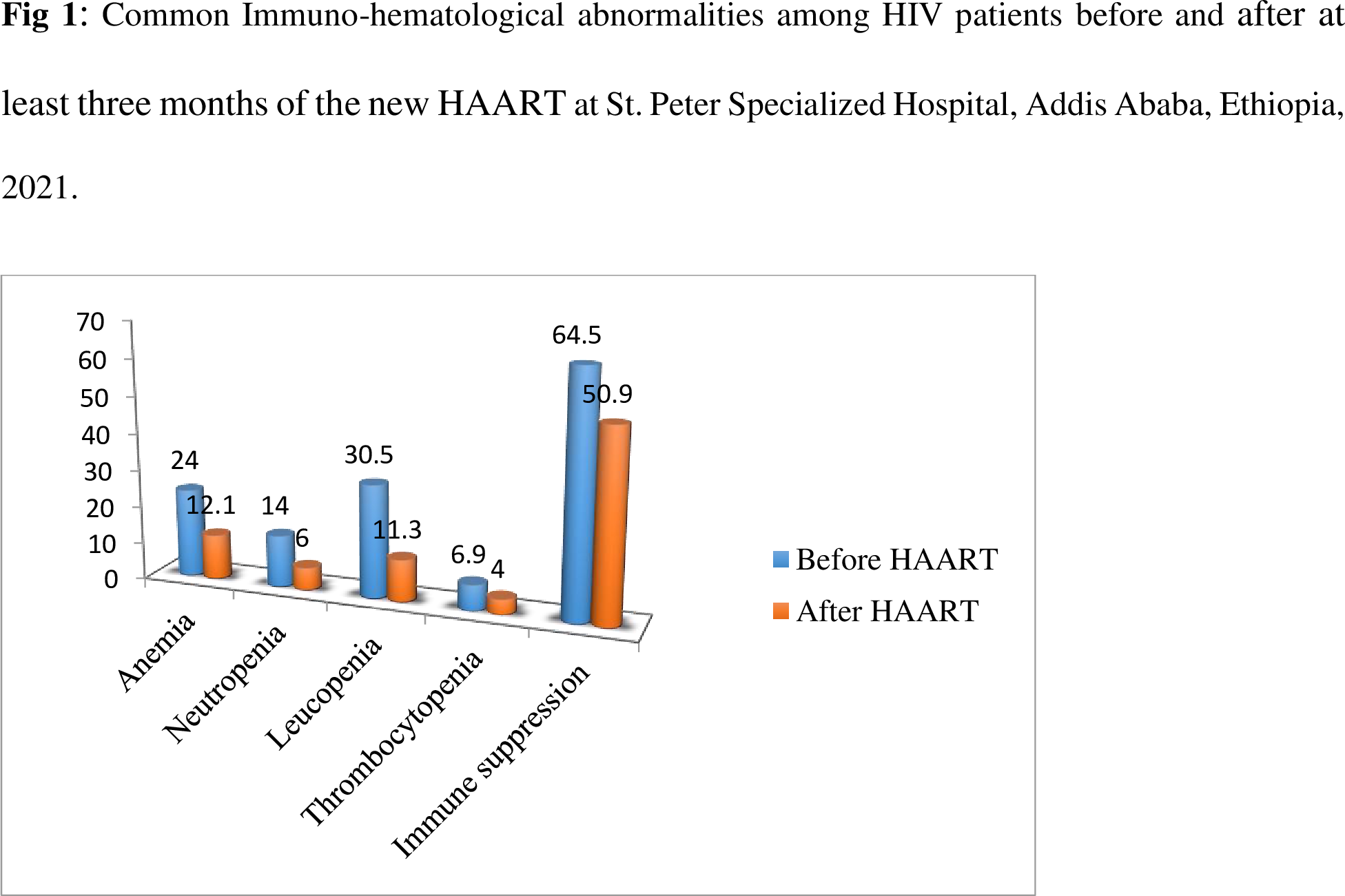

Supplement: S1 Fig — (TIF) [file pone.0310239.s002.tif]
